# Subtype-specific differences in the risk of hospitalisation among patients infected with hepatitis E virus genotype 3 in Belgium, 2010–2018

**DOI:** 10.1017/S0950268819001122

**Published:** 2019-06-24

**Authors:** Lorenzo Subissi, Michael Peeters, Sophie Lamoral, Sofieke Klamer, Vanessa Suin, Steven Van Gucht

**Affiliations:** 1 National Reference Centre of Hepatitis Viruses, Sciensano, Brussels, Belgium; 2 European Public Health Microbiology Training Programme, European Centre for Disease Prevention and Control, Stockholm, Sweden; 3 Epidemiology of Infectious Diseases, Sciensano, Brussels, Belgium

**Keywords:** Food-borne infections, hepatitis, infectious disease epidemiology, molecular epidemiology, zoonotic foodborne diseases

## Abstract

Some European countries recently reported an increase in hepatitis E virus genotype 3 (HEV-3) of the subtype 3c. No link between HEV-3 subtypes and severity is established to date. Here, we report that patients infected with HEV-3c were at lower risk of hospitalisation, compared to those infected with HEV-3f, the other main subtype circulating in Belgium.

## Introduction

Hepatitis E virus (HEV) is the most common cause of acute hepatitis worldwide [1] with increasing numbers of autochthonous cases being reported across Europe [2]. These cases are mainly related to genotype 3 (HEV-3), which is further sub-grouped into three major clades (3abchij, 3efg and 3ra) [3, 4]. Clinical manifestations related to HEV-3 range from unapparent to symptoms of hepatitis of varying severity, with few reported cases of fatal outcomes. It is unknown whether the different HEV-3 clades and/or subtypes differ in the severity of symptoms they can provoke.

In different EU countries, an increasing number of HEV-3c human infections were reported [5–8]. In Belgium, the recent increase of 3c translated to an even distribution of subtypes 3c and 3f, the most common representatives of the two HEV-3 major clades, providing Belgium as an optimal setting to study the differences in severity between more distantly related HEV-3 subtypes [8]. A minority of patients were also diagnosed with HEV-1, thought to be travel related because of the similarities of the isolated sequences with non-European HEV-1 variants, and with HEV-4, closest to sequences isolated from France [8].

Here, we compared information on hospitalisation status-as reported in the request form for hepatitis E virus testing sent to the National Reference Centre (NRC)-in patients infected (i) with different HEV genotypes and (ii) with different HEV-3 subtypes. The aim is to better understand the clinical relevance and impact of the different hepatitis E viruses circulating in Belgium and inform future, bigger, multi-country studies.

## Methods

From 1 January 2010 until 30 June 2018, we retrieved information for patients with HEV infection confirmed at the NRC. The information regarded hospitalisation status, age, gender and sequencing results. The submission of samples to the NRC is on a voluntary basis. Any Belgian general practitioner/ clinician from any hospital or peripheral laboratory may send serum samples to the NRC. The NRC performs primary diagnosis for both HEV serology and/or HEV RNA. In addition, primary laboratories may send positive and equivocal samples to the NRC for confirmation diagnosis and surveillance purposes. During the study period, 86% of the primary clinical laboratories in Belgium provided at least one HEV-suspected sample to the NRC for diagnosis and genotyping. For the purpose of surveillance, quantitative Real-Time polymerase chain reaction (qRT-PCR) is not only done upon request but also on all IgM positive samples, even if not requested by the clinician, and subtyping is done on all qRT-PCR positive samples. Patients were included in the study if they had a HEV RNA positive sample, tested using the commercial RealStar^®^ HEV RT-PCR Kit 1.0 (Altona Diagnostics, Germany) and were successfully genotyped as described in Suin *et al*. [8]. We used two sets of degenerated HEV-specific primers for the RT-PCR assay, adapted from Huang *et al*. [9]. External primers used were: 3156N (forward, 5′-AATTATGCC(T)CAGAC(T)CGG(A)GTG-3′) and 3157N (reverse, 5′-CCCTTA(G)TCC(T)TGCTGA(C)GCATTCTC-3′); internal primers were: 3158N (forward, 5′-GT(A)ATGCTT(C)TGCATA(T)CATGGCT-3′) and 3159N (reverse, 5′-AGCCGACGAAATCAA TTCTGC-3′). We used this protocol, which aimed at sequencing 348 base pairs of the ORF2 region, between 2010 and 2016. Since 2017, we changed to the protocol described by Boxman *et al*. [10], which aimed at sequencing 493 base pairs of the same region (ORF2).

We performed a descriptive as well as uni- and multi-variable analyses using log-binomial regression (or robust Poisson regression when the first did not converge) to estimate risk ratios. The main outcome of the study was hospitalisation status and the main exposures were (i) HEV genotype and (ii) HEV-3 clades or main Belgian subtypes. Variables with *P* < 0.2 in the univariable analysis were retained for the multivariable analysis. Trends over time of hospitalisation rates were analysed using a non-parametric test for trend across ordered groups (Cuzick extension of the Wilkoxon rank sum test). Data were analysed using STATA v.14.

## Results

During the study period, we received 10 942 samples from suspected patients. Of those, 523 were HEV positive (188 IgM positive/PCR negative and 335 PCR positive). Genotyping was successful for 80% of PCR-positive samples (269/335). Of those, 32 had missing data on hospitalisation, two on age and one on gender and were excluded from the study. Of those with missing data for hospitalisation, 19 were infected with HEV-3f, eight were infected with HEV-3c, three were infected HEV-3e and one was infected with HEV-1. A total of 234 qRT-PCR-positive patients diagnosed with HEV at the NRC were included in the study.

Patient characteristics are reported in Table 1. The median age of patients in the study was 59 years (interquartile range, IQR, 50–66). The median age was 60 (IQR 50–66) for genotype 3, 62 (IQR 55–74) for genotype 4 and 43 (IQR 32–52) for genotype 1 infections. HEV-3c accounted for 90% of clade 1 sequences (90/100) and HEV-3f for 87% of clade 2 sequences (92/106). HEV-3e, the third most common HEV-3 subtype, accounted for 10% of clade 2 sequences. The other subtypes identified in Belgium were: HEV-3l (formerly known as 3f), 4% of clade 2 sequences (4/106); HEV-3h, 3% of clade 1 sequences (3/100); HEV-3a, 2% of clade 1 sequences (2/100); HEV-3i, 1% of clade 1 sequences (1/100). A new subtype, closely related to clade 1, accounted for 4% of clade 1 sequences (4/100).

**Table 1.** Characteristics of study participants (*N* = 234)

	*N*	Percentage
Hospitalisation		
Yes	98/234	58
No	136/234	42
Age (years)		
0–29	13/234	6
30–49	44/234	19
50–64	108/234	46
⩾65	69/234	29
Sex		
Female	65/234	28
Male	169/234	72
HEV genotype		
HEV-1	13/234	6
HEV-4	7/234	3
HEV-3	214/234	91
HEV-3c	90/214	42
HEV-3e	10/214	5
HEV-3f	92/214	43
Other HEV-3 subtypes	15/214	7
Unassigned HEV-3	7/214	3
--------------------------		
Clade 1 (3abchij)	100/214	47
Clade 2 (3efg)	106/214	50
Clade 3 (3ra)	1/214	0
Unassigned clade	7/214	3

Compared to HEV- 3 patients, HEV-1 patients had twice the risk of hospitalisation (adjusted risk ratio (aRR) 2.0, 95% confidence interval (CI) 1.6–2.5), after adjusting for age (Table 2). When restricting the analysis to HEV-3 infected patients, the hospitalisation risk was 1.7-times higher for patients belonging to clade 3efg, as compared to patients belonging clade 3abcjhi (aRR 1.7, 95% CI 1.2–2.4). Similarly, when we compared the three main Belgian subtypes, patients infected with HEV-3e or 3f were at higher risk of hospitalisation, compared to patients infected with HEV-3c (respectively aRR 2.3, 95% CI 1.2–4.4; aRR 1.6, 95% CI 1.1–2.3, Table 3). Hospitalisation rates did not change over time (non-parametric test for trend *P* = 0.70)

**Table 2.** Adjusted risk ratios and 95% CI for the association of hospitalisation status and age with HEV genotype (*N* = 234)

	Outcome: hospitalisation status			
Independent variables	Crude RR	95% CI	aRR^a^	95% CI
Age				
<65	Ref		Ref	
⩾65	1.3	1.0–1.8	1.5	1.2–1.9
Sex				
Female	Ref		Ref	
Male	1.1	0.8–1.5	1.0	0.7–1.4
HEV genotype				
Genotype 3	Ref		Ref	
Genotype 1	1.5	1.0–2.4	2.0	1.6–2.5
Genotype 4	1.4	0.7–2.8	1.2	0.6–2.6

a Adjusted for all other variables in the table.

**Table 3.** Adjusted risk ratios and 95% CI for the association of hospitalisation status and age with (a) HEV-3 clades (*N* = 206) and (b) main Belgian subtypes (*N* = 192). Unassigned HEV-3 (*n* = 7) and clade 3 (3ra, *n* = 1) were excluded from model A and model B, and other HEV-3 subtypes (*n* = 14) were excluded from model B.

	Outcome: hospitalisation status			
	Crude RR	95% CI	aRR^a^	95% CI
*Model A*				
Age				
<65	Ref		Ref	
⩾65	1.3	1.0–1.8	1.5	1.2–2.4
Sex				
Female	Ref		Ref	
Male	1.1	0.8–1.5	1.0	0.7–1.4
HEV-3 clades				
Clade 1 (3abchij)	Ref		Ref	
Clade 2 (3efg)	1.6	1.1–2.2	1.7	1.2–2.4
*Model B*				
Age				
<65	Ref		Ref	
⩾65	1.3	1.0–1.8	1.5	1.1–2.2
Sex				
Female	Ref		Ref	
Male	1.1	0.8–1.5	1.0	0.7–1.5
HEV-3 main Belgian subtypes				
3c	Ref		Ref	
3e	2.0	1.1–3.6	2.3	1.2–4.4
3f	1.5	1.0–2.2	1.6	1.1–2.3

a Adjusted for the other variables in the table.

## Discussion

In this study, we found a lower risk of hospitalisation at the time of diagnostic request among patients with HEV-3c infection, compared to HEV-3f, the other main subtype circulating in Belgium. We also found an increased risk of hospitalisation for HEV-1-infected patients, as compared to HEV-3-infected ones. This is in line with studies pointing at higher morbidity associated with HEV-1 [11], which uses different routes of transmission than HEV-3 and HEV-4.

Smith *et al*. compared the subtype distributions of HEV-3 infected blood donors (asymptomatic) and HEV-3 patients (symptomatic) in four different European countries, but they did not report any statistically significant association. [3]. Because they compared blood donors with symptomatic HEV patients-and did not use any information on disease outcome among the latter group-their results cannot be directly compared to the ones presented here. Moreover, the heterogeneity of information that was used, coming from different countries, with different HEV surveillance systems in place, may have also played a role. Despite these limitations, some of their analyses pointed towards mild/asymptomatic infections being possibly more frequent in individuals with clade 1 (3abchij) infections, as opposed to clade 2 (3efg) infections (*P*-value<0.1) [3].

A limitation of the present study is that the information on hospitalisation is partially based on the request forms that the NRC receives when a HEV test is requested. The criteria for hospitalising a patient may depend on the GP and/or hospital. Another limitation is that information on co-morbidities or other clinical details was not available and may, therefore, have a role of confounding factors that we could not take into account. In fact, older patients with underlying liver disease may be at higher risk of developing more severe hepatitis E [1]. PCR-positive HEV patients for whom genotyping was not successful, or for whom hospitalisation status, age or gender were not available, had to be excluded from the analysis. This may have led to selection bias, though it is unlikely that the distribution of missing data was significantly associated with the HEV genotype/subtype.

Robust HEV-3 infectivity assays will be needed to confirm differences in the disease severity associated with HEV-3 clades/subtypes and study the molecular mechanisms behind those differences. Meanwhile, we recommend that clinicians be aware of the increasing HEV burden in Belgium and the heterogeneous disease severity that hepatitis E viruses can cause.

## Transparency declaration

The manuscript's guarantor affirms that the manuscript is an honest, accurate and transparent account of the study being reported; no important aspects of the study have been omitted; any discrepancies from the study as planned have been explained.

## Data sharing

N/A

## Ethics approval

The study falls under the official mandate of the National Reference Centre for Hepatitis Viruses to collect and analyse epidemiological and clinical data of hepatitis E cases in Belgium as part of the national surveillance plan and promotion of public health. Considering the retrospective and non-interventional nature of the study, approval of an ethics commission or individual informed consent is not required, in agreement with the Belgian Law of the 7 May 2004 concerning experiments on people.
